# Detection of H_2_S, HF and H_2_ pollutant gases on the surface of penta-PdAs_2_ monolayer using DFT approach

**DOI:** 10.1038/s41598-023-27563-x

**Published:** 2023-01-13

**Authors:** Dhara Raval, Sanjeev K. Gupta, P. N. Gajjar

**Affiliations:** 1 Department of Physics, University School of Sciences, Gujarat University, Ahmedabad, 380009 India; 2 Computational Materials and Nanoscience Group, Department of Physics and Electronics, St. Xavier’s College, Ahmedabad, 380009 India

**Keywords:** Electronic properties and materials, Electronic properties and materials

## Abstract

In this research, the adsorption of targeted noxious gases like H_2_S, HF and H_2_ on penta-PdAs_2_ monolayer are deeply studied by means of the density functional theory (DFT). After the capturing of three kind of pollutant gases (H_2_S, HF and H_2_), it is observed that, the electronic properties are slightly affected from the pristine one. In all cases, the physisorption interaction found with adsorption energy of − 0.49, − 0.39 and − 0.16 eV for H_2_S, HF and H_2_ gases, respectively. Which is exposed that H_2_S gas strongly absorbed on penta-PdAs_2_ nanosheet. In case of HF (H_2_) gas adsorbed systems, the obtained charge transfer is + 0.111 e (+ 0.037 e), revealed that the electrons are going to PdAs_2_ nanosheet from the HF (H_2_) molecules. Further, under the non-equilibrium Green’s function (NEGF) theory, the IV response and sensitivity of absorbed H_2_S, HF and H_2_ have been discussed. The results demonstrate that the H_2_S molecules on PdAs_2_ has suitable adsorption strength and explicit charge transfer compared with other targeted molecules. Hence, our novel findings of H_2_S, HF and H_2_ targeted gas sensing on penta-PdAs_2_ nanosheet might provide reference-line to design modern gas sensor device at the nano-scale.

## Introduction

H_2_S, HF and H_2_ are toxic and colorless gases, and they are common industrial and environmental pollutants, and also became extensive range sources of health effects^1–3^. Additionally, due to rapid industrialization, universally use of chemicals, transportation of automobiles has led to increasing global environmental pollution and diminution of the ozone layer, which has expected a great hazard and major threat to the human health^4–6^. However, the arena of gas sensing has always concentrated on the research in control of environmental pollution, monitoring of industrial activities, health diagnostics and noxious gas sensing’s sake of public safety^7–9^. In recent years, numerous nanomaterials have been scrutinized for gas sensing applications. In spotlight, the two-dimensional (2D) nanomaterials have castoff to sense a low concentration of gas molecules in sensing applications because of their compensations like, high stability, novel structures, large surface area/volume ratio, excellent adsorption abilities and low cost^10–19^. For example, graphene^20^, Phosphorene^21^, carbon nitride compound^22^, B_4_C_3_ monolayer^23^, InN monolayer^24^ and transition-metal dichalcogenides (TMDs) like MoSe_2_, PtSe_2_, SnS_2_ and HfS_2_, etc.^25–30^, have become the burgeoning materials as toxic gases sensor.

Moreover, the key foundations of (i) H_2_S gas are from volcanic gas, fuel, sewage plants, sulfur deposits and ammonia synthesis from hydrocarbon feedstock^31–37^. Although, very recently, a study shows B_4_C_3_ nanosheet, as excellent gas sensor for CO, NO, NH_3_, SO_2_ and H_2_S^23^ and Gao et al.^38^, testified the strong adsorption capacity of H_2_S and CH_4_ with the modified 2D graphene sheet. Then, (ii) HF gas is also eminent toxic gas it is mainly originate from industrial processes at high temperature and the combustion of products containing fluoride, which is harmfully affect to not even only human but also flora and fauna. The high exposure of HF gas causes, muscle spasms and result may even in fatality at extreme cases^2^.

Bhattacharya et al.^38^, also studied the sensing of HF, HCN and H_2_S with nitrogenated holey carbon (C_2_N-*h*2D) monolayer. Next, (iii) H_2_ a tasteless, odourless and flammable gas. Even, by lacking O_2_ concentration in enclosed area, only 4% concentration of H_2_ gas could be reasons to burnt and suffocation^39^. Li et al.^40^ also explored Ru-doped PtSe_2_ monolayer as good sensor of H_2_ gas and Pandey et al.^41^ also suggested that MoS/WS monolayer is potential substrates for gas sensing of H_2_, NO and CO gases.

Apart from aforementioned two-dimensional (2D) materials gas sensor, the newly discovered pentagonal structures had been also showed to design gas sensor due to its superior quality such as high surface carrier mobility, large surface area, more adsorption sites and great optical properties over the traditional unit hexagon structure^42,43^. Additionally, in past, several 2D pentagon unit structure has also been reported for the promising candidates for innovative applications in the gas sensor. Like, Wei et al.^44^ predicted that penta-BCN monolayer is good sensor of CO, H_2_S, NH_3_ and NO gas molecules and stated that the targeted gases have moderate adsorption energies in the range of − 0.797 eV to − 1.186 eV and proved it as potential applicant for said gas molecules sensors applications. Lakhani et al.^45^ shows the dissociation of air pollutants on the uniform surface of pentagonal BeP_2_ monolayer using first principles study. Xia et al.^46^ also explored that penta-PdSe_2_ monolayer has meaningful and promising material to be applied in FET type gas sensors as detection of NO_2_ gas. Then, Tang et al.^47^ revealed the new sensor material P-SiC_2_ monolayer and concluded that after the contact with NO_2_ gas the electronic resistance of P-SiC_2_ monolayer is decreased significantly, which indicating the ultrahigh sensitivity towards NO_2_ sensing. Hereafter, inspired by these Penta two-dimensional (2D) gas sensing research work owing to their robust adsorption capabilities and sensitivity, herein we are first time examined H_2_S, HF and H_2_ gas sensing capabilities on the newly discovered novel penta-PdAs_2_ monolayer, because modelling new gas sensing resources have paramount bid to probe the pollutant molecules in the current era. Viz, H_2_S, HF and H_2_ gases. And finding an effective material to detecting and adsorbing these toxic (H_2_S and HF) and highly flammable (H_2_) gases are great worth to protect our living environment for modern civilization. Even though, the outstanding electronic properties, transport, and optical properties of pristine penta-PdAs_2_ monolayer is previously done by our research group^48^.

In this work, the capacity of monolayer penta-PdAs_2_ as gas sensor (of H_2_S, HF and H_2_ gases) capture is discovered by first-principles calculations. As density functional theory calculation is a widespread and feasible approach for simulation of gas adsorption on the novel nano-surfaces. Henceforth, the adsorption process of H_2_S, HF and H_2_ gases on penta-PdAs_2_ monolayer was studied and compared by evaluating differences in adsorption energy (*E*_*ad*_), appearance change (*e*), charge transfer (*Q*), work function (*Φ*), charge density difference (CDD), electronic band structure, density of states/eV (DOS) and partial density of states/eV (PDOS) in this research note. These observations deliver quest of sensor materials with high sensitivity and selectivity towards pollutant gases such like H_2_S, HF and H_2_.

## Simulation details

**Figure 1:**
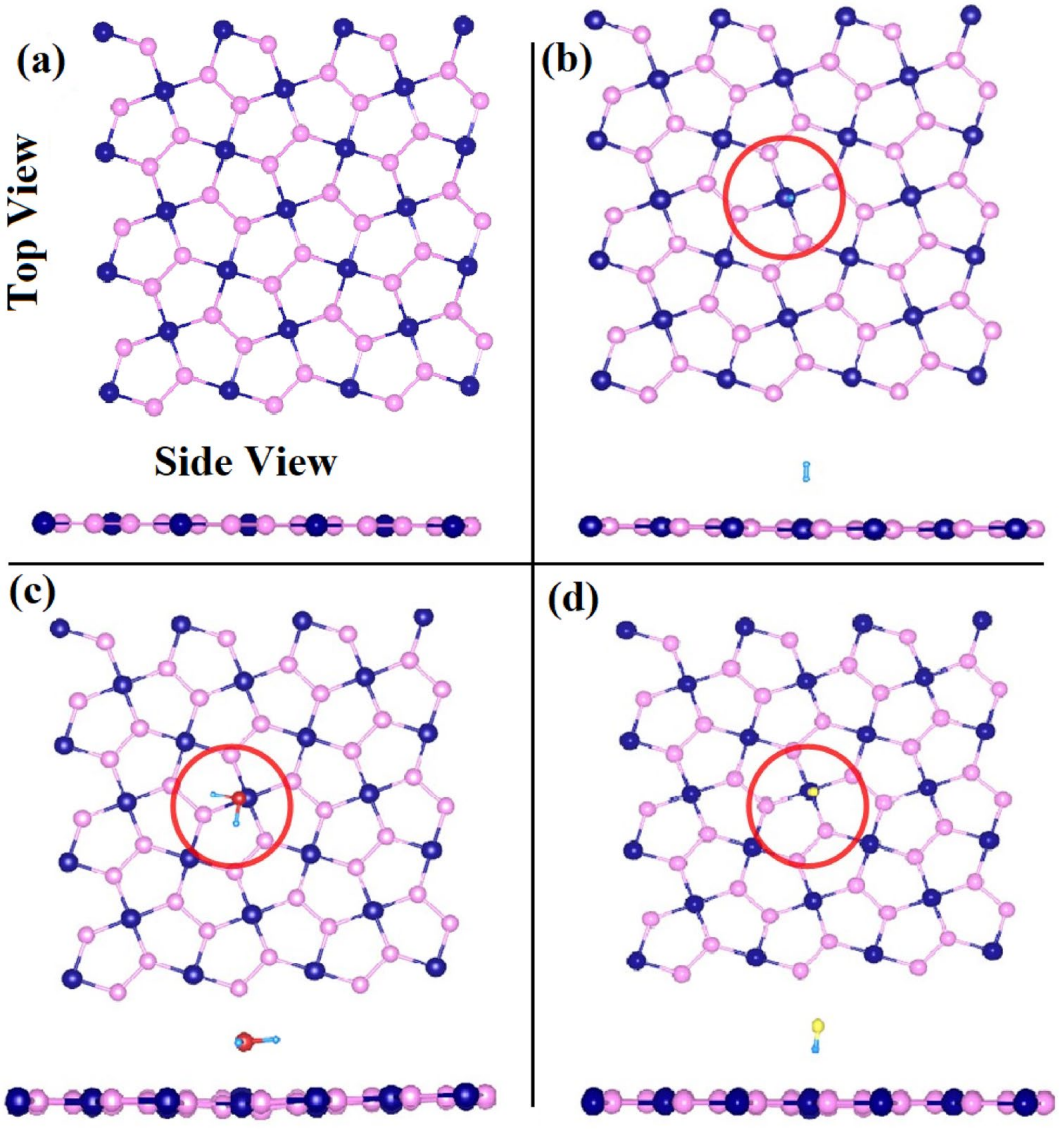
Side and top view of the (**a**) Bare penta-PdAs_2_ monolayer and after gas adsorption of (**b**) H_2_ (**c**) H_2_S and (**d**) HF. The Pd, As, H, S and F atoms are indicated in navy blue, pink, water blue, red and yellow colors, respectively.

Density Functional theory (DFT) calculations were executed using SIESTA code^49^ to examine the optimization, electronic and adsorption (of targeted gases) properties of penta-PdAs_2_ nanosheet. To treat the exchange–correlation (XC) effect, the generalized gradient approximation (GGA) of the Perdew–Burke–Ernzerhof (PBE)^50^ functional was employed. We incorporated the *DRSLL* dispersion correction method to treat the long range *vdWs* forces^51^. The Kohn–Sham one electron states were expanded in a plane-wave basis set. The plane wave kinetic energy cut-off is considered as 450 Ry. The doubled zeta plus (*DZP*) basis set was used with an energy of 0.01 Ry to expand the Kohn–Sham orbital. The Brillouin zone sampled using a 5 × 5 × 1 Monkhorst–Pack k-point mesh during structural optimization^52^. The **k**-point mesh was then increased to 15 × 15 × 1 to attain more accurate results for electronic structure simulations. The optimized structures of 3 × 3 × 1 supercell penta-PdAs_2_ monolayer is shown in Fig. 1a. As aforementioned, potential properties of penta-PdAs_2_ monolayer such as structural, electronic, transport and optical properties have already reported by our group^48^ and proven that it is dynamically stable via the positive phonon frequencies of penta-PdAs_2_. Hence, using same substrate of penta-PdAs_2_, herein we reported the sensing potential in the penta-PdAs_2_ monolayer for H_2_S, HF and H_2_ gases. All the optimized structures were relaxed without given any geometric constraint (GC) on the sheet. The atomic force is specified as 0.01 eV/Å to relaxing the systems. A vacuum of 15 Å have been considered along the perpendicular direction of the penta-PdAs_2_ surface to avoid the interaction of periodic boundary conditions as employed in XY-plane. The adsorption energy is determined by the following equation^53^:

1 $$E_{ads} = E_{{{\text{nanosheet}} + {\text{Gas}}}} {-}E_{{{\text{nanosheet}}}} - E_{{{\text{Gas}}}} ,$$

where, *E*_nanosheet+Gas_ represents the total energy of the penta-PdAs_2_ nanosheet with the gas molecules absorbed on, while *E*_nanosheet_ and *E*_Gas_ are the total energy of penta-PdAs_2_ sheet without the gas molecules and the isolated gas molecules, respectively. From the adsorption energy definition, a negative E_ads_ specifies an exothermic process and thermodynamically favourable for molecular gas sensing. All the initial distances between sheet and gas molecules are set to 2.0 Å. The Hirshfeld atomic population analysis method was used to estimate the charge transfer (*Q*) as defined in SIESTA and negative value of *Q* means that electron transfer sheet to gas molecules. The charge density difference (CDD) Δ*ρ* between the adsorbed and isolated gases is calculated by:

2 $$\Delta \rho = \rho_{{{\text{nanosheet}} + {\text{Gas}}}} - \rho_{{{\text{nanosheet}}}} - \rho_{{{\text{Gas}}}} ,$$

where, ρ_nanosheet+Gas_, ρ_nanosheet_ and ρ_Gas_ are represent the charge densities of penta-PdAs_2_ monolayer with, without the adsorption of gas and charge density of gas molecules, respectively. This CDD has been imagined with utility of VESTA software^54^. The recovery time (τ) is the crucial parameter for gas sensor, which can be measured experimentally ranging from second to several minutes. Based on activated-complex theory^55^, recovery time has been defined by:

3 $${\uptau = \omega }^{-1}\mathrm{exp}\left(-\frac{{E}_{ads}}{{K}_{B}T}\right),$$

where, *ω* is the attempt frequency (~ 10^12^ s^−1^), *E*_*ads*_ is adsorption energy, *K*_*B*_ is Boltzmann’ Constant and *T* is temperature. At last, based on non-equilibrium *Green’s* function (NEGF), the IV response was examined by TRANSIESTA package^56^. The current (I) through the scattering region is calculated by exploring the Landauer-Buttiker formalism,

4 $$I\left({V}_{bias}\right)={G}_{0}{\int }_{{\mu }_{L}}^{{\mu }_{R}} T\left(E,{V}_{bias}\right) \left[f \left(\mathrm{\rm E}-{\mu }_{L}\right)-f \left(\mathrm{\rm E}-{\mu }_{R}\right)\right] dE,$$

where, *G*_0_ is quantum conductance and *T* (*E*, *V*_*b*_) is the transmission probability of an electron incident at an energy *E* through the device under the bias voltage *V*_*bias*_, ${\mu }_{L}$ and ${\mu }_{R}$ are the electrostatic potentials of left and right electrodes.

## Results and discussions

### Adsorption of H_2_, H_2_S and HF gases on penta-PdAs_2_ monolayer

In the first step, we have systematically examined the geometries of pristine penta-PdAs_2_ monolayer. The optimized structure of penta-PdAs_2_ is displayed in Fig. 1a. The bond length between Pd–As and As–As are 2.51 Å and 2.32 Å, respectively, which are comparable with previous reported studies^57^. To study the electronic properties such as the electronic band structure, DOS and PDOS were calculated and the obtained electronic band gap (*E*_*g*_) of penta-PdAs_2_ is 0.34 eV^48^. In the second step, we have optimized the gas molecules of H_2_S, HF and H_2_ on the surface of penta-PdAs_2_ sheet. We have placed the gas molecules at all possible parking sites on the sheet and initially molecules are fixed at the (i) Hollow (ii) Top of Pd atom (iii) Top of As atom (iv) Bridge of Pd-As and (v) Bridge of As-As atoms. The adsorption energy of each case is reported in ESI (Table S1, ESI) and highest adsorption energy site is considered for the further calculations. Therefore, the most favorable sites of H_2_S and H_2_ gases are perceived at bridge of Pd-As with adsorption distance of 2.91 Å and 2.70 Å, respectively. While HF gas is relaxed on the top of Pd atom with distance of 2.34 Å. Although, after the relaxation of that sites, the gas molecules are shifted on the top of Pd atom in all cases, which is shown in Fig. 1b–d. After the stable adsorption, the bond length between H–S, H–H, and H–F are 1.37, 0.77 and 0.94 Å, respectively and the bond angle of non-linear H_2_S is 92.142°, which is agreed with previous reported work^58^.

Table 1 presents the adsorption energy (*E*_*ads*_), adsorption distance (*d*) and relaxation time (*τ*) and type of interaction for considered H_2_S, HF and H_2_ gas molecules on the surface of penta-PdAs_2_ monolayer. As shown in table, the calculated adsorption energy of H_2_S, HF and H_2_ gases are − 0.49, − 0.39 and − 0.16 eV, respectively. The *E*_*ads*_ values of H_2_S, HF and H_2_ were negative, suggesting that the adsorption process was all energetically favorable on the penta-PdAs_2_ monolayer and all in the physisorption range. Among three gases, H_2_S (HF) has strongest with 0.49 (0.39) eV compared to H_2_, it has the weakest adsorption strength about *E*_*ads*_ ~ 0.16 eV. Although, the obtained adsorption energy of H_2_ is 18.75% higher compared to adsorbed on phosphorene (0.13 eV) nanosheet^59^. Additionally, Majidi et al. reported the HF and H_2_S detection on the twin graphene and Ti-embedded twin graphene with quoted adsorption energy of − 0.16 and − 0.22, respectively^58^. Which means, present results are quite good compared to them as H_2_S and HF gas sensor on penta-PdAs_2_ sheet. On other side, the obtained adsorption energy of H_2_S gas on penta-PdAs_2_ monolayer is − 0.49 eV, which is smaller than penta-BCN monolayer (− 0.797 eV)^44^, but relatively larger than functionalized graphene^58^ and phosphorene (− 0.41 eV)^21^. Further, it also be noted that the recovery time (*τ*) plays a crucial role in sensing application, *τ* is also summarized in the Table 1. The obtained sequence of the recovery time (τ) for three gases are as follow: H_2_ < HF < H_2_S. Although, the recovery time of H_2_S is very long owing to the strong adsorption capacity of PdAs_2_ + H_2_S (0.49 eV). Hence, the low value of recovery time strongly specified that PdAs_2_ + H_2_S configuration have high selectivity and can be good choice as reversible sensors of H_2_S.

**Table 1:** The adsorption energy (*E*_*ads*_), adsorption distance (*d*), relaxation time (*τ*) and interaction type.

System	*E*_*ads*_ (in eV)	*d* (Å)	*τ* (in µs)	Interaction type
PdAs_2_	–	–	–	–
PdAs_2_ + H_2_	− 0.16	2.70	0.00053	Physisorption
PdAs_2_ + H_2_S	− 0.49	2.91	179	Physisorption
PdAs_2_ + HF	− 0.39	2.34	4.24	Physisorption

**Figure 2:**
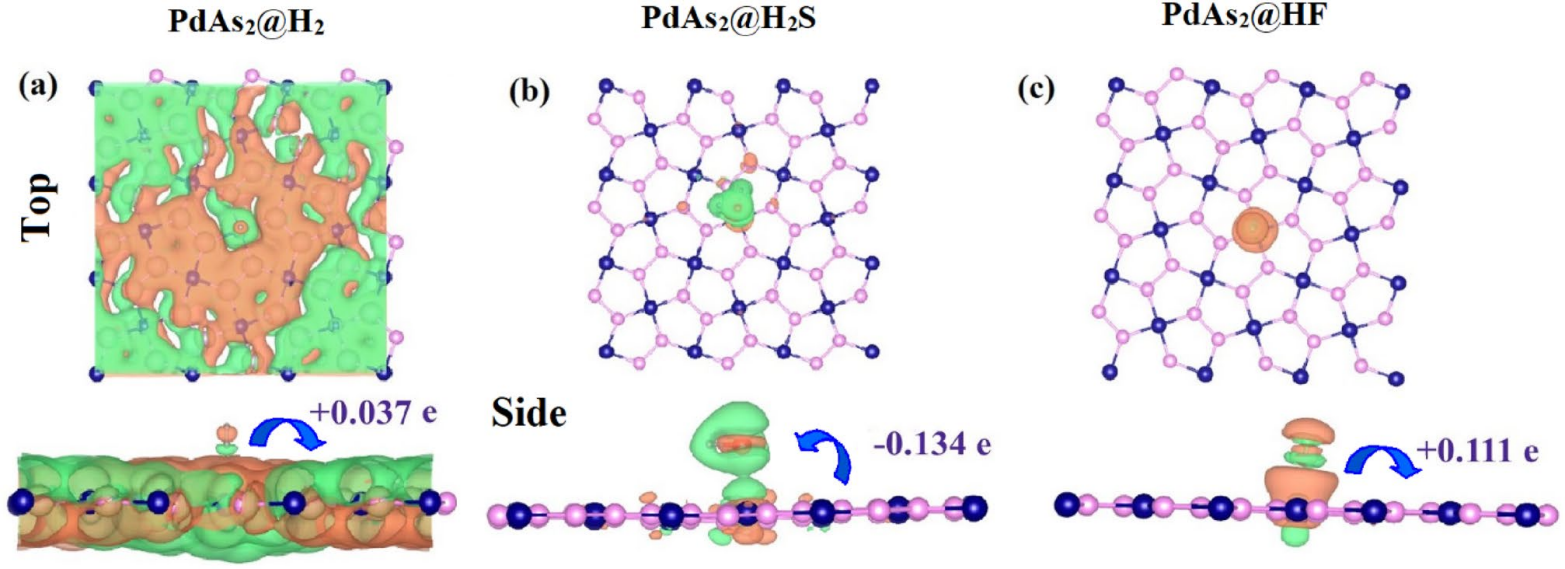
The charge density difference (CDD) and charge transfer of (**a**) H_2_, (**b**) H_2_S and (**c**) HF molecules after absorbed. Green and orange color represent accumulation and depletion of charge, respectively. The isovalue used for plotting is 0.03 e/Å^3^.

Moreover, the charge transfer mechanism was studied by Hirshfeld atomic population analysis and results are summarized in Table 2. The value of Q < 0 indicates that H_2_S (− 0.137 e) gas molecules accept the electrons from the PdAs_2_ sheet. whereas Q > 0, in HF (+ 0.111) and H_2_ (+ 0.037) indicates that both gas molecules are donating electrons to the PdAs_2_ sheet as result of that holes are expected to stay in the gas molecules as depicted in Fig. 2 by the blue arrow. Figure 2a–c, shows the charge density difference (Δρ), which is obtained by the Eq. (2). From that, it can be observed that, charge is accumulated at the negative charge region (shown as green color) and charge is depleted in the positive charge region (shown as orange color) due to the reformed of the effective interface dipoles and polarization of electrons. Where adsorption energy (*E*_*ads*_) is taking place to form the redistribution of electrons at the interface region. Here, among the three gases, the H_2_S has higher charge transfer between the adsorbing PdAs_2_ sheet suggesting that H_2_S has effective and strong detection with high selectivity towards the penta-PdAs_2_ nanosheet compared to the HF and H_2_ gases.

**Table 2:** The electronic band gap (*E*_*g*_), charge transfer (*Q*), work function (*Φ*) and style.

System	*E*_*g*_ (eV)	*Q* (*e*)	*Φ* (eV)	Style
PdAs_2_	0.34	–	4.631	–
PdAs_2_ + H_2_	0.30	+ 0.037 e	4.634	Donor
PdAs_2_ + H_2_S	0.28	− 0.134 e	4.573	Acceptor
PdAs_2_ + HF	0.30	+ 0.111 e	4.708	Donor

**Figure 3:**
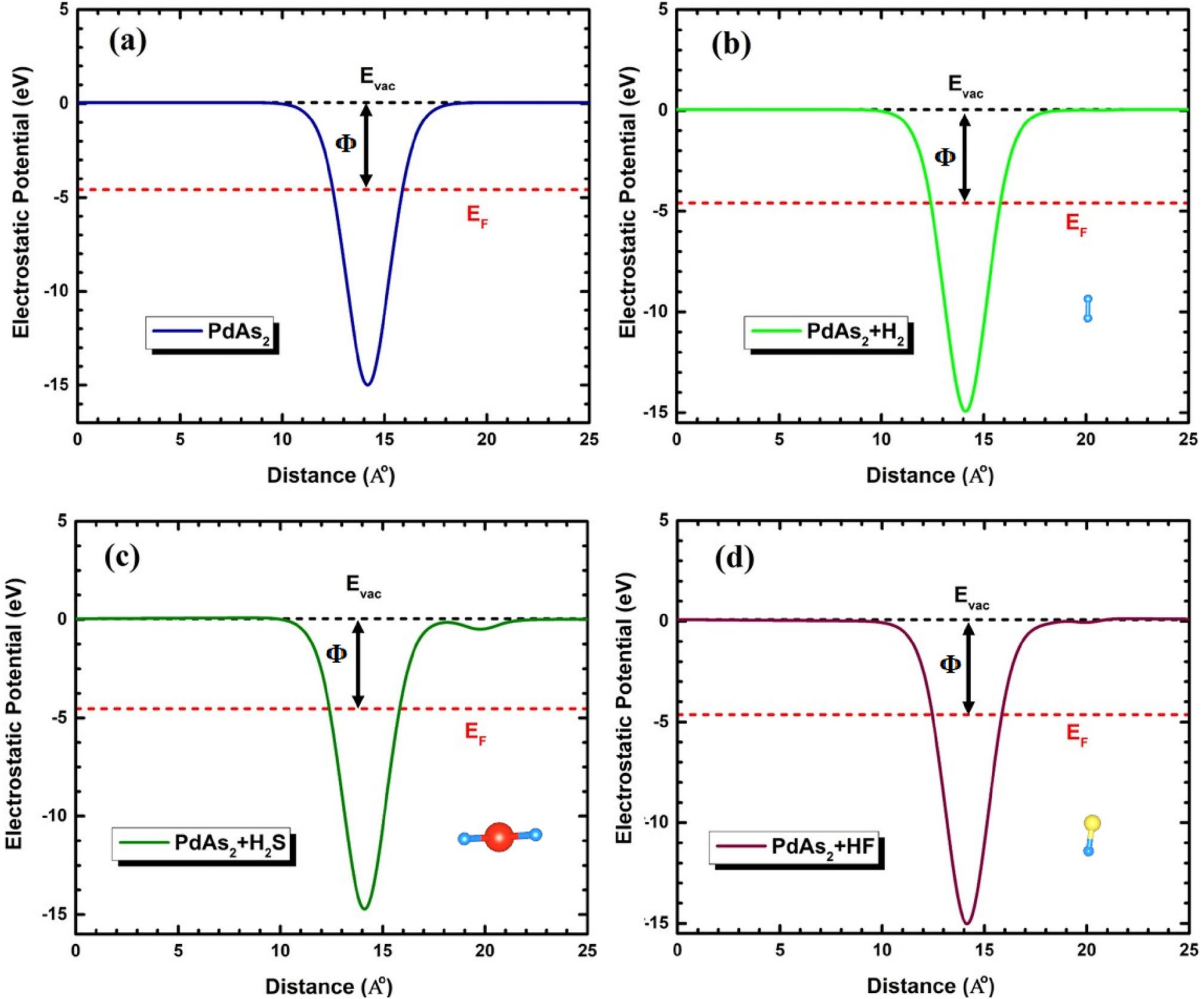
The planar average of the electrostatic potential of (**a**) bare monolayer and adsorbed with (**b**) H_2_, (**c**) H_2_S and (**d**) HF gas molecule.

The surface sensitivity of the 2D nanosheet could also approached by determining the differences in work function parameter (Φ)^60^ as illustrate:

5 $$\Phi \, = \, V_{\infty } - \, E_{F} ,$$

where, *V*_*∞*_ and *E*_*F*_ are the electrostatic potential and the fermi energy level, respectively. Basically, the work function describes the minimum energy needed to dislodge an electron from the surface of penta-PdAs_2_ monolayer. The calculated work function of penta-PdAs_2_ with H_2_S, HF and H_2_ lies in the range of 4.63 to 4.70 eV, as tabulated in Table 2 and electrostatic potential energy level is presented in in Fig. 3a–d. The red and black dashed line indicates the fermi energy level (*E*_*F*_) and energy vacuum level (*V*_*∞*_), respectively and the black double head arrow gives the work function values. The value of *Φ* of bare graphene is 4.5 eV which shows it as an ideal material for where work function optimization is vital^61^. As shown in Table 2, the *Φ* of bare penta-PdAs_2_ monolayer is 4.63 eV that is slightly larger than the graphene^61^. The nature of the *Φ* is symmetric for H_2_S, HF and H_2_ gas molecules with *Φ* value of 4.573 eV, 4.708 eV and 4.634 eV, respetively. Because of the adsorption of gases, the *Φ* is increase in all the cases compared to bare PdAs_2_ monolayer demonstrating that the electrons are transferring to the vacuum level is obstructed. Therefore, the *Φ* can be effectively affected by the adsorption of H_2_S, HF and H_2_ gas molecules on PdAs_2_ surface, which implies that Schottky barrier height can be adjusted by the H_2_S, HF and H_2_ gas molecules.

### Electronic properties

**Figure 4:**
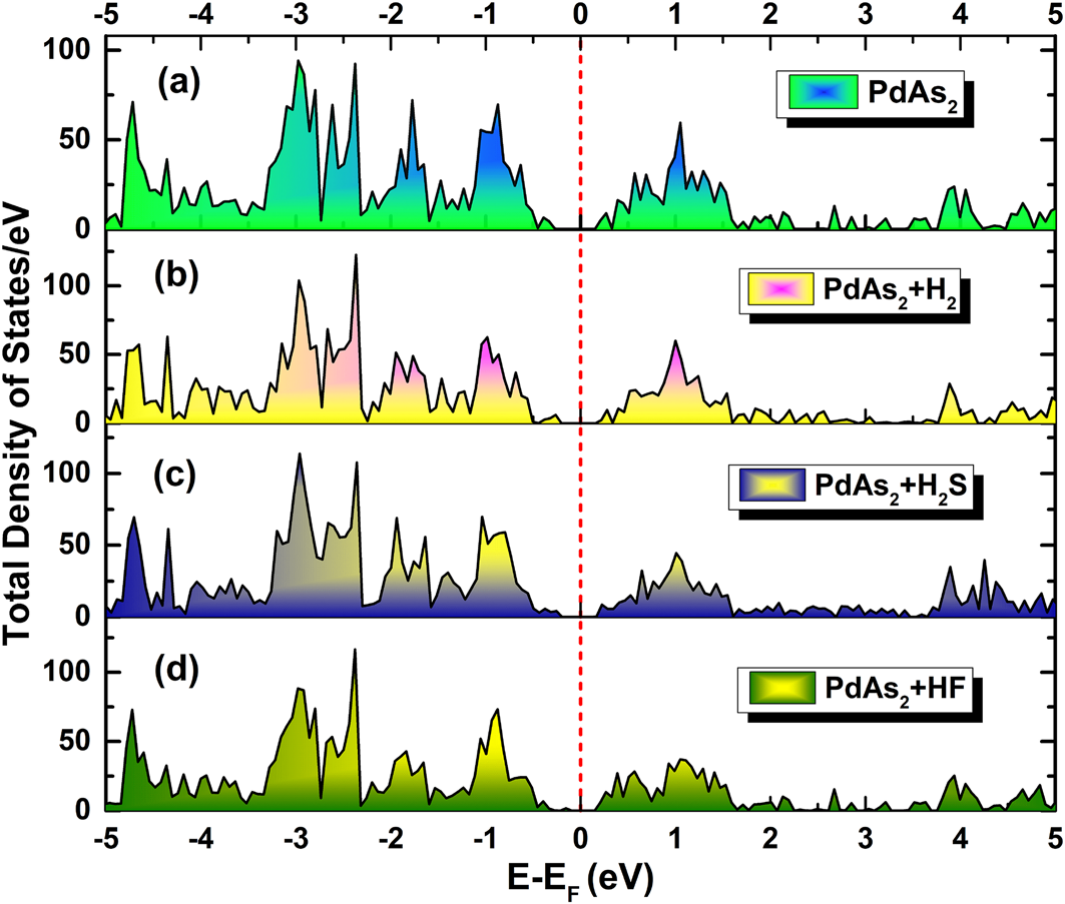
Total density of states/*eV* of (**a**) penta-PdAs_2_ with adsorbed (**b**) H_2_, (**c**) H_2_S and (**d**) HF gas molecules.

In order to deeper elucidate the gas sensing behavior of penta-PdAs_2_ monolayer towards H_2_S, HF and H_2_ gases, we analyzes the total density of states/eV (TDOS), projected density of states/eV (PDOS) and electronic band structure of gas molecules absorbed along that of bare PdAs_2_ monolayer system. Figure 4a–d, shows the density of state before and after the adsorption of H_2_S, HF and H_2_ gases on the PdAs_2_ surface. For comparison, the electronic band struture and TDOS and PDOS also demostrated in the ESI (Figs. S1, S2, ESI).

Visibly, it is confirmations no significant change either the electronic conduction or valence bands in the band structure after the adsorption of the gas molecules but the electronic band gap (*E*_*g*_) is decreases from the bare counterparts. As tabulated in Table 2, the *E*_*g*_ decreases to 0.28 and 0.30 as a result of H_2_S and HF(H_2_) adsorption on pristine PdAs_2_ monolayer, respectively. The trivial impact of H_2_S, HF and H_2_ molecules in the unoccupied states of the PdAs_2_ monolayer above the fermi level is highly responsible to decreases the electronic band gap due to π to π* transition at S-point after the adsorption of the gases. Additionally, the valance bands (VBs) and conduction bands (CBs) have new sub-bands are outcome from the adsorption of H_2_S, HF and H_2_ gases on PdAs_2_ monolayer. The presence of new sub-bands justifies the variations of the electronic energy levels due to the adsorption of three gases. Therefore, TDOS/eV and PDOS/eV in further utilized to investigate the energy levels of bare PdAs_2_ prior and after adsorption of H_2_ H_2_S and HF as shown in Fig. 4a–d. It can be observe from Fig. 4a–d, the significant contribution in the DOS comes from the Pd-*d* and As-*p* orbitals with a small contribution from H, S and F atoms at the fermi level. From the DOS of the H_2_ adsorption (Fig. 4b), one can see that PdAs_2_ monolayer after adsorbing upholds the semiconducting feature with electronic band gap of 0.30 eV and H_2_ gas molecules acts as electron donor. However, there is no more influence seen at fermi level between PdAs_2_ + H_2_ and bare PdAs_2_ monolayer. But, the *1s* orbital of H atom in the H_2_ is mainly hybridizied with the *4p* orbital of the As atom and localized between 5 to 6 eV in the conduction bands as shown in Fig. S2a. While in case of H_2_S absorption (Fig. 4c), the highest occupied molecules orbital (HOMO) was mainly dominated by the interrelation of the H-*1s* and S-*3p* orbital of the H_2_S gas molecules and induces distinct states around 3.5 to 4.5 eV in the CBs. The same trend also follows in the adsorbing of H_2_S gas molecules on the CoOOH sheet surface^62^. The relatively higher adsoprtion energy and charge transfer of H_2_S on the PdAs_2_ sheet surface was due to the hybridyzation between the Pd-*4d* and S-*3p* states near the fermi level in the VBs as depicted in Fig. S2b. Similarly, after the adsorption of the HF gas molecules (Fig. 4d), the energy around 4 to 6 eV is arised due to the mixing of the H-*1s* and F-*2p* orbital in the HOMO region. While strong contribution of the hybridization of the F-*2p* (− 4 to − 2 eV) orbitals and Pd-*4d* are responsible for the next higher adsorption energy of the PdAs_2_ + HF system and acts as the electron donor near the fermi energy level as viewed in Fig. S2c. Likewise, very recently, Kaur et al. also reported the 2D janus WSSe monolayers as efficient nanosensor towards toxic HF gas molecules and the PDOS of adsorbed HF gas molecules is also contributed as same as here presented results^63^. Therefore, the remarkable ability of penta-PdAs_2_ monolayer makes a potential candidate for the H_2_S gas sensing applicatons.

### Electric transport properties of adsorbed H_2_S, HF and H_2_ gases on penta-PdAs_2_ monolayer

**Figure 5:**
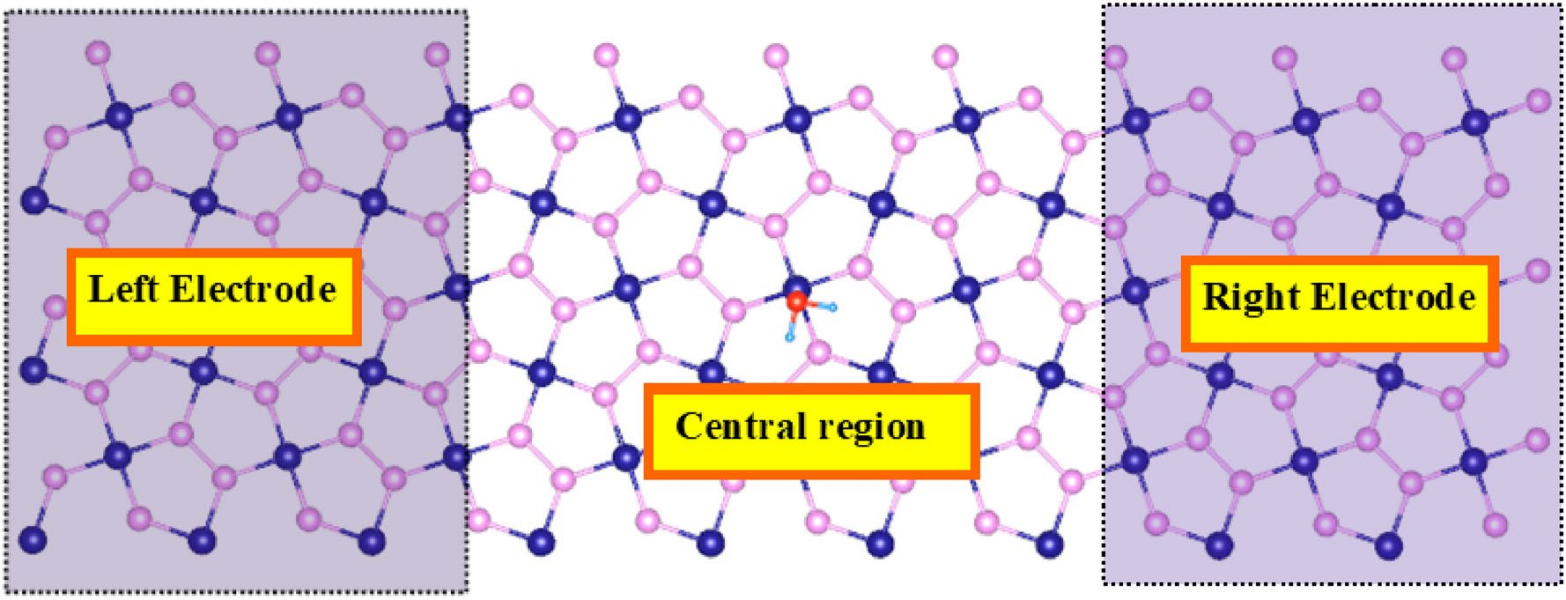
Schematic structural model of a gas sensor based on penta-PdAs_2_ + H_2_S with two electrodes. The Pd, As, H and S atoms are indicated in navy blue, pink, water blue and red colors, respectively.

**Figure 6:**
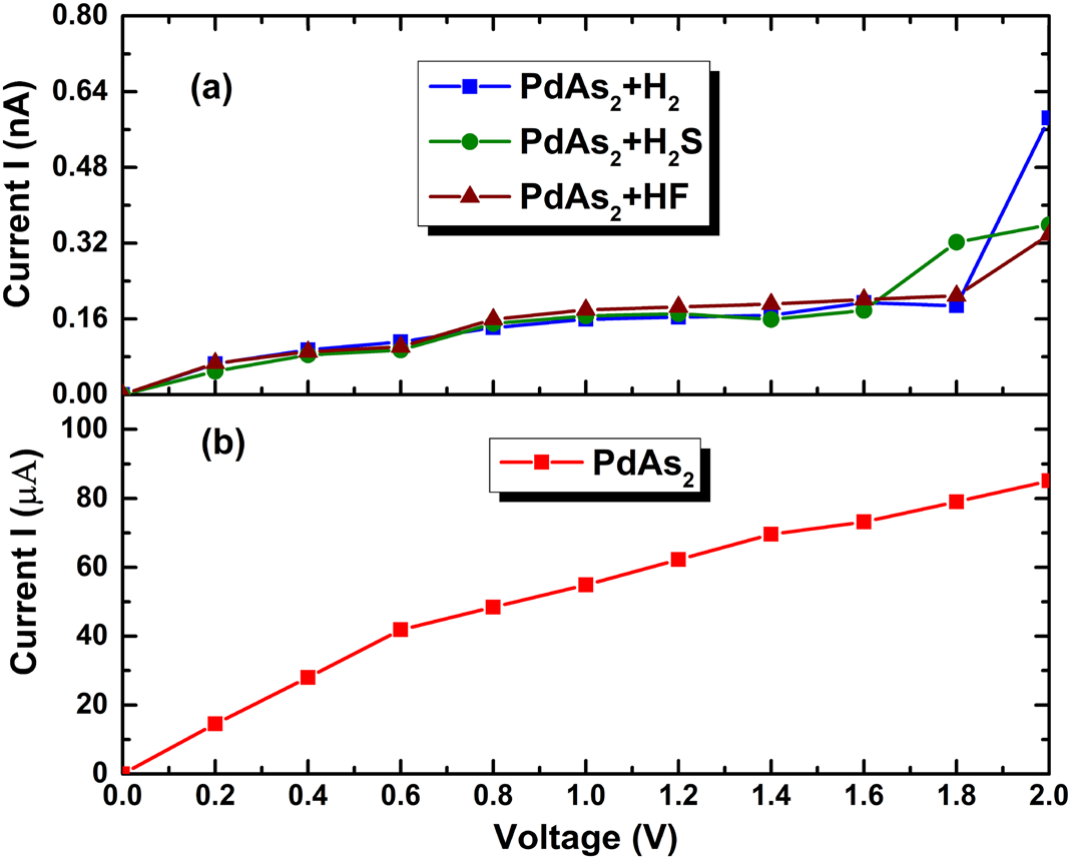
The current–voltage charateristics of adsorbed (**a**) H_2_, H_2_S and HF gas molecules, (**b**) bare PdAs_2_ monolayer.

To quantitatively probe the gas sensing properties of the PdAs_2_ being an efficient nano sensor, non-equilibrium *Green’s* function formalism (NEGF) was employed to analyze the current (I)-voltage (V) response^64^. The structural schematic model of sensing device set up are shown in Fig. 5. Where, the shaded region shows the electrodes which were simulated within the a 3 × 3 supercell of bare PdAs_2_ monolayer. The central or scattering region exemplifies the device area, where gas adsorption take place. Figure 6a,b, exhibits the I–V characteristics of PdAs_2_ before and after gas molecules exposure.

Although the adsorption of H_2_S, HF and H_2_ gas molecules does not affect much the electronic structure pointedly, but the charge transfer (*Q*) after their exposure is expected to vary due to doping of electrons or holes during the electron transportation. And these effects are revealed to be possible indication for gas sensors. As shown in Fig. 6b, In the case of bare PdAs_2_, we found that the current response in the range of micro-ampere (µA). Further, the junction remains switched off at the 0 V but seems switched on with the increasing of bias voltage. Then, noticeably above the 0 V*,* all the absorbed gas systems have a relevant effect on the transport from the bare one, which making all systems for gas sensing applications.

As seen in Fig. 6a, after the gas exposure the current is reducing from the bare PdAs_2_ and gives response in order of nA. When bias over 0 V, the current starts to increase simultaneously up to 1.6 V for all cases. Interestingly, after the bias of 1.6 V the current response trends change slightly for all absorbed gas molecules. At V = 1.8 V, the highest current sensitivity (current of 0.32 nA) with current is observed for the PdAs_2_ + H_2_S system compared to HF and H_2_ system. Which could be due to acceptor nature of the H_2_S gas molecules. Further, in the H_2_ adsorption (Fig. 6a), the current is significantly enhanced and reached up to 0.58 nA at the bias of 2 V. While it calibrate, for H_2_S (with current of 0.35 nA) and HF (with current of 0.33 nA) gas exposure a current reduction is about 60% and 56.89%, respectively compared to H_2_ gas exposure under the bias of 2 V as summarized in the Table 3. Overall, all the results indicated that the penta-PdAs_2_ monolayer could be better applicants for achieving the sensitivity and selectivity towards H_2_S compare to HF and H_2_ gas molecules.

**Table 3:** The current ratio and current variation of H_2_, H_2_S and HF absorbed PdAs_2_ nanosheet at the bias voltage of 2.0 V.

System	Current at bias 2.0 V		Current variation (with adsorbed H_2_) (%)
	Current I	Current ratio I_R_	
PdAs_2_ nanosheet	85 µA	1 µA	–
PdAs_2_ + H_2_	0.58 nA	6.82 µA	100
PdAs_2_ + H_2_S	0.35 nA	4.11 µA	60
PdAs_2_ + HF	0.33 nA	3.88 µA	56.89

## Conclusion

In summary, the noxious H_2_S, HF and H_2_ gas molecules on a penta-PdAs_2_ nanosheet are first time investigated within the density functional level of theory including van der Waals corrections. We systemically discovered the most favorable binding site of each molecule based on the five different adsorption positions. The adsorption energy (*E*_*ads*_), charge transfer (*Q*), recovery time (*τ*) and work function (*Φ*) were investigated to understand performance and behavior of adsorption of the pollutant on PdAs_2_ surface. The gas sensitivity order of pollutant gases was predicted as follow: H_2_S > HF > H_2_ on the penta-PdAs_2_ nanosheet. The obtained recovery time (*τ*) of the sensor to all target gases are less than 3 min. The adsorption of H_2_S and HF results in an obvious increasing in DOS near E_F,_ which is not observed by the adsorption of H_2_. Eventually, the *IV* response were carried out to study the responses of bare and absorbed PdAs_2_ nanosheet and the sharp rises seen at V = 1.8 V in order of H_2_S > HF > H_2_ that shows the authenticated potential as efficient gas sensor. These outcome acclaim the exciting prospects of developing penta-PdAs_2_ monolayer for the ultrahigh-sensitivity gas sensing nano-devices.

## Supplementary Information

Supplementary Information.

## Data Availability

The datasets generated and/or analysed during the current study are not publicly available due to privacy or other restrictions. However, it may be made available from the corresponding author on reasonable request.
