# Supplementary material for: Detection of H2S, HF and H2 pollutant gases on the surface of penta-PdAs2 monolayer using DFT approach
Source: Sci Rep. 2023 Jan 13;13:699. doi: 10.1038/s41598-023-27563-x (PMC9839685; doi:10.1038/s41598-023-27563-x)
Supplement: Supplementary file 1 — Supplementary Information. [file 41598_2023_27563_MOESM1_ESM.docx]

**Supplementary Materials**

**Detection of H_2_S, HF and H_2_ pollutant gases on the surface of Penta-PdAs_2_ Monolayer Using DFT Approach**

Dhara Raval^1^, Sanjeev K. Gupta^2, *^ and P. N. Gajjar^3,^ **^*^**

^1,3^Department of Physics, University School of Sciences, Gujarat University, Ahmedabad 380009, India.

^2^Computational Materials and Nanoscience Group, Department of Physics and Electronics, St. Xavier's College, Ahmedabad 380009, India.

**Keyword (s):**

^*^Corresponding authors: Prof. (Dr.) P. N. Gajjar (E-mail: pngajjar@gujaratuniversity.ac.in, pngajjar@rediffmail.com) and Dr. Sanjeev K. Gupta (E-mail: sanjeev.gupta@sxca.edu.in)

| **Site** | **H_2_S** | **HF** | **H_2_** |
| --- | --- | --- | --- |
| **Hollow** | -0.490 | -0.3838 | -0.1340 |
| **Top of Pd** | -0.4912 | -0.3950 | -0.1613 |
| **Top of As** | -0.4908 | -0.3412 | -0.1340 |
| **Bridge of Pd-As** | -0.4918 | -0.3507 | -0.1625 |
| **Bridge of As-As** | -0.4236 | -0.3438 | -0.1315 |

Table S1 : The adsorption energy of H_2_S, HF and H_2_ at the different position site on penta-PdAs_2_ monolayer. The Cyan color indicates the highest energy of each gases, which means that site is most preferable for the adsorption of the gas molecules.


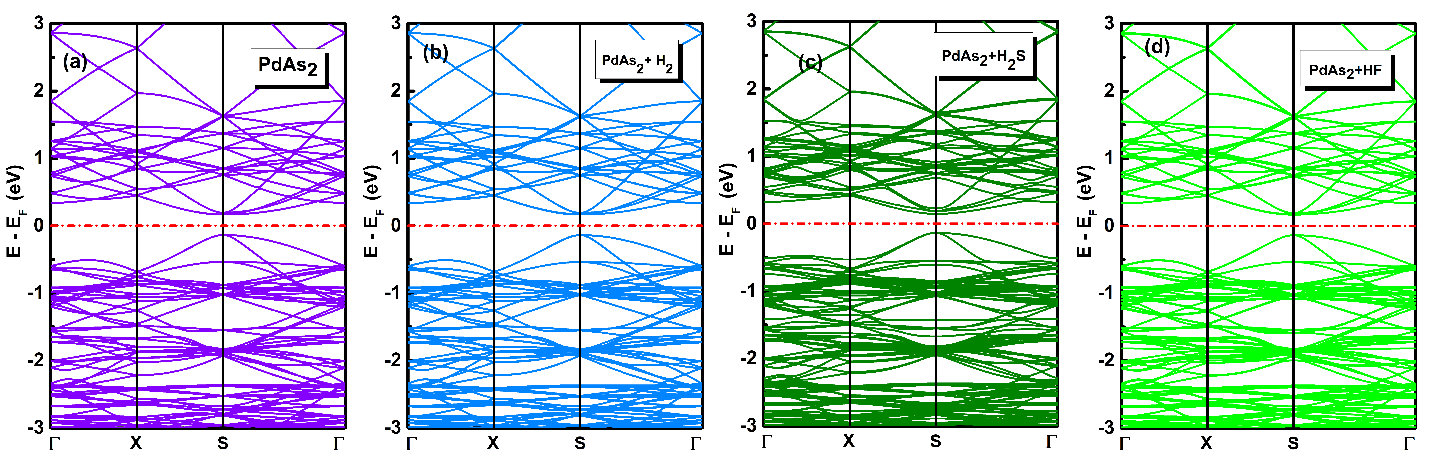


**Figure S1** : Electronic band structure of (a) Bare (b) H_2_ absorbed (c) H_2_S absorbed (d) HF adsorbed Penta-PdAs_2_ sheet.


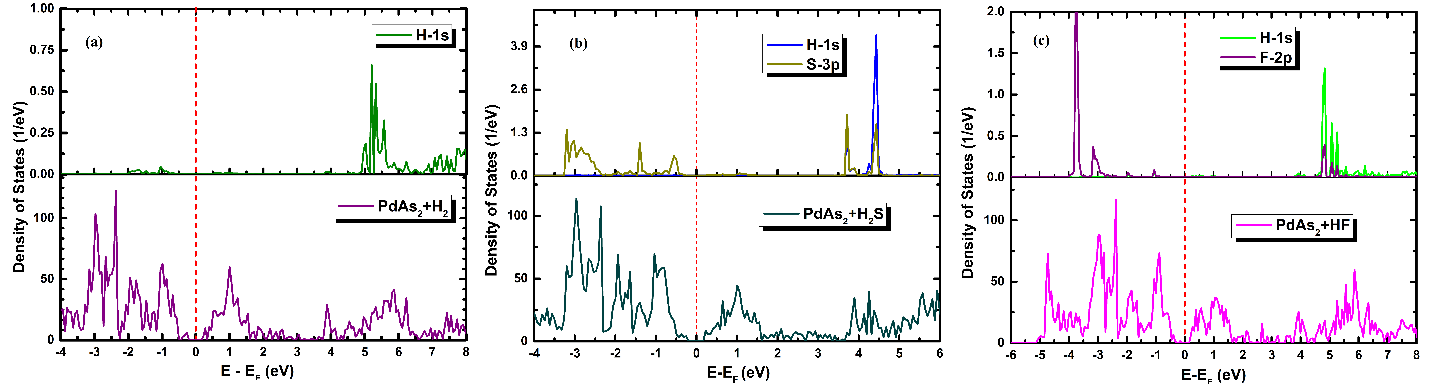


**Figure S2 :** Partial density of states of (a) H_2_ absorbed (b) H_2_S absorbed (c) HF adsorbed Penta-PdAs_2_ sheet.
